# Predictive value of the Distress Thermometer score for risk of suicide in patients with cancer

**DOI:** 10.1007/s00520-022-06801-4

**Published:** 2022-02-24

**Authors:** Yung-Chih Chiang, Jeremy Couper, Jing-Wen Chen, Ke-Jui Lin, Han-Ping Wu

**Affiliations:** 1 Department of Psychiatry, Kaohsiung Veterans General Hospital, Kaohsiung, Taiwan; 2 Eastern Health, Melbourne, Victoria Australia; 3 Department of Psychiatry, Monash University, Melbourne, Victoria Australia; 4 Department of Nursing, College of Medicine and Life Science, Chung Hwa University of Medical Technology, Tainan, Taiwan; 5 Cancer Center, Kaohsiung Veterans General Hospital, Kaohsiung, Taiwan; 6 Department of Pediatric Emergency Medicine, Children’s Hospital, China Medical University, Taichung, Taiwan; 7 Department of Medical Research, Department of Pediatric Emergency Medicine, Children’s Hospital, China Medical University, No. 2, Yuh-Der Road, Taichung, Taiwan; 8 Department of Medicine, School of Medicine, China Medical University, Taichung, Taiwan

**Keywords:** Cancer, Distress Thermometer score, Suicidal ideation, Suicide, Death

## Abstract

**Purpose:**

This study aimed to assess the association between the Distress Thermometer (DT) score and risk of suicide in patients with cancer. In addition, we aimed to determine the best cutoff score to predict patients at risk of suicide.

**Methods:**

From 2015 to 2016, we retrospectively collected data on patients with cancer. DT scores were collected, and the association between DT score and risk of suicide (suicide ideation or death ideation) was analyzed. Furthermore, receiver operating characteristic (ROC) analysis was performed to identify the appropriate cutoff score for predicting risk of suicide.

**Results:**

A total of 260 patients with cancer were included, and suicidal ideation was identified in 33 cases referred for psychological intervention. The DT scores of the patients with suicidal ideation were significantly higher than those of patients without suicidal ideation (6.30±2.11 vs. 4.29±1.72, *p*<0.05). In addition, the area under the ROC curve for predicting risk for suicide was 0.758. The cutoff DT score of 3 had the highest sensitivity of 1.00 to rule out suicidal ideation, while 9 had the highest specificity of 1.00 to rule in suicidal ideation. Moreover, the appropriate cutoff DT score to predict patients with suicidal ideation was 5, with a sensitivity of 0.52, specificity of .84, positive likelihood ratio of 3.24, and negative likelihood ratio of 0.58.

**Conclusion:**

The DT score may be a helpful clinical tool to evaluate emotional distress and risk of suicide in patients with cancer. Clinically, for DT scores greater than 5 in patients with cancer, the risk of suicide greatly increases. In view of the DT’s widespread use internationally by non-mental health clinicians in cancer to guide the need for specialist mental health interventions, its potential utility in also predicting suicide risk is of great interest.

## Introduction

Cancer patients often experience significant distress and may be at a higher risk of suicide than the general populations and other patients [1–4]. The suicide rates among cancer patients are approximately twice compared with general population [5]. The intensity of suicide intent progresses from death ideation to suicidal ideation and suicidal behavior [5–8]. Death ideation is defined as having a wish to die without thought of taking one’s own life [9]. The presence of death ideation or suicidal ideation is associated with increased risk for suicide [10]. Early detection of suicidal ideation or death ideation may help primary caregivers to prevent suicide in patients with cancer. In addition, systematic screening for suicidal ideation is recommended; however, screening for suicidal ideation is not be commonly implemented in routine clinical practice in the cancer setting [11].

It is known that suicidal ideation in patients with cancer may be related to their physical and psychological distress [12, 13]. The National Comprehensive Cancer Network (NCCN) recommends screening for distress in patients with cancer and thus developed the Distress Thermometer (DT) scoring system, a well-known tool using a 0–10 rating scale [14]. DT is commonly used by primary oncology team, and referral to mental health professionals is considered if patient’s DT score meets the cutoff point. Although DT is widely used in screening distress in cancer patients, as far as our understanding, only one study examined the correlation between DT scores and suicidal ideation, and revealed higher DT scores predict higher risk of suicidal ideation [15].

In this study, we aimed to identify whether the DT score may be helpful for initially screening the risk of suicide in patients with cancer, and we assessed the association between the DT score and risk of suicide (suicidal ideation or death ideation) in a sample of patients with cancer. Furthermore, we identified a recommended cutoff score for predicting patients at risk of suicide.

## Methods

We retrospectively reviewed the medical records of patients with cancer referred to clinical psychologists for intervention at Kaohsiung Veterans General Hospital in Taiwan from January 2015 to Dec 2016. The exclusion criteria included patients who refused to undergo assessments and where patients were assessed to have significantly impaired cognitive status.

This study was approved by the Institutional Review Board and Ethics Committee of Kaohsiung Veterans General Hospital, and all procedures were performed in accordance with the relevant guidelines and regulations. Data were retrospectively reviewed, obtained, de-identified, and anonymized before analysis, and the Ethics Committee waived the requirement for informed consent because of the anonymized nature of the data and scientific purpose of the study.

Clinical information related to age, gender, initial diagnosis (versus recurrence), cancer type, types of treatment, and record of psychologists’ assessment were obtained from the medical records. For the referred patients with cancer, the psychologists conducted a semi-structured interview, evaluated and recorded routinely their chief complaint, mental and physical status, support system, DT scores, and suicide risk, including suicidal ideation and death ideation. Suicidal ideation was defined as the patient’s idea or plan to take their own life by certain means; while death ideation indicated that patients made clear their desire of not wanting to live, hoping their life would end sooner but not mentioning the idea of taking their own life by certain means. Based on the assessments of psychologists regarding patients’ suicide risk, patients were divided into the three groups: (1) suicidal ideation, (2) death ideation (without suicidal ideation), and (3) neither suicidal ideation nor death ideation (“no risk”).

### Statistical analysis

The association of patient characteristics and DT score with risk of suicide was analyzed. The chi-square test, Fisher’s exact test, Student’s *t*-test, Mann-Whitney *U* test, analysis of variance (ANOVA), and Games-Howell post hoc test were used as appropriate. In the descriptive analysis, values were presented as means ± standard deviations (SDs). Differences between groups were presented with 95% confidence intervals.

In addition, receiver operating characteristic (ROC) curve analysis was used to identify the appropriate cutoff score to predict risk of suicide. The test characteristics of the different cutoff values, namely sensitivity, specificity, area under the ROC curve (AUC), positive likelihood ratio (LR^+^), and negative likelihood ratio (LR^-^), were also examined. The AUC, calculated using the trapezoidal rule, was considered to be a standard measure of the diagnostic value of the parameter. An optimal test result had a value of 1.0, while 0.5 was not considered to be useful. The LR^+^ and LR^-^ were calculated for the best cutoff values. The criterion value indicated the value corresponding to the highest accuracy (minimal false negative and false positive results). Statistical significance was set at *p*<0.05. All statistical analyses were performed using SPSS software (version 22.0; SPSS Inc., Chicago, IL, USA).

## Results

During the period from January 2015 to Dec 2016, a total of 22,190 cancer patients were hospitalized in Kaohsiung Veterans General Hospital. Out of 22,190 patients, 394 patients (1.78%) with cancer were referred to the hospital’s psychologists for evaluation. For the purpose of this study, 134 patients were excluded because of incomplete data (lacking DT scores or suicide assessment data) or impaired cognitive status. Therefore, a total of 260 patients with cancer were included in the analysis.

Among the 260 patients, 33 had suicidal ideation, and 20 had death ideation. The mean age was higher in the death ideation group compared with the suicidal ideation and no-risk groups (*p*<0.05) (Table 1). The average DT score was 6.3±2.1 for the 33 patients with suicidal ideation, and 5.2±1.4 for the 20 patients with death ideation. However, the average DT score of the 207 no-risk patients was 4.3±1.7, which was lower than that of the other two groups. The average DT score was highest in the suicidal ideation group compared to the death ideation and no-risk groups. Because of unequal sample sizes and unequal variances, Games-Howell post hoc test was used to analyze the difference of DT scores between 3 groups. The DT scores between suicidal ideation group and no-risk group and those between death ideation group and no-risk group showed significant differences (*p*<0.05). However, the DT scores between suicidal ideation group and death ideation group are not statistically significant (*p*>0.05).

**Table 1:** Demographics of cancer patients according to suicide risk

Variables	Suicidal ideation (*n*=33)	Death ideation (*n*=20)	No risk (*n*=207)	*P* value
Age (years) M±SD (95% CI)	58.1±17.1 (52~64.12)	61.4±12.4 (55.62~67.18)	53.3±13.9 (51.44~55.25)	0.018
DT score M±SD (95% CI)	6.3±2.1 (5.55~7.05)	5.2±1.4 (4.50~5.80)	4.3±1.7 (4.05~4.52)	<0.001
Gender (male), *n* (%)	17 (51.5)	9 (45)	98 (47.3)	0.877
Initial diagnosis, ***n* (%)**	30 (90.9)	17 (85)	193 (93.2)	0.397
Type of cancer, *n* (%)				
Gastric & esophageal	3(9.1)	1 (5)	17 (8.2)	
Breast	2 (6.1)	1 (5)	15 (7.2)	
Lymphoma	3 (9.1)	1 (5)	31 (15)	
Lung	3 (9.1)	2 (10)	15 (7.2)	
Gynecologic	4 (12.1)	3 (15)	17 (8.2)	
Colon	6 (18.2)	0	28 (13.5)	
Pancreatic	0	0	2 (1)	
Head and neck	4 (12.1)	4 (20)	21 (10.1)	
Leukemia	1 (3)	0	15 (7.2)	
Hepatoma	1 (3)	4 (20)	13 (6.3)	
Bladder	1 (3)	0	4 (1.9)	
Other	5 (15.2)	4 (20)	29 (14)	
Type of treatment, ***n* (%)**				
Nil	12 (36.4)	4 (20)	45 (21.7)	
Chemotherapy	17 (51.5)	13(65)	126 (60.9)	
Surgery	3 (9.1)	1 (5)	29 (14)	
Radiotherapy	0	1 (5)	4 (1.9)	
Hospice care	0	1 (5)	2 (1)	
Other	1 (3)^a^	0	1 (0.5)^b^	

^a^Surgery + chemotherapy

^b^Percutaneous ethanol injection (PEI)

Among the different cancer types, the proportion of patients with death and suicidal ideation was higher in those with head and neck cancers, and lower in patients with lymphoma and leukemia. Moreover, among the types of treatment, most patients with suicidal ideation and death ideation were receiving chemotherapy.

**Fig. 1:**
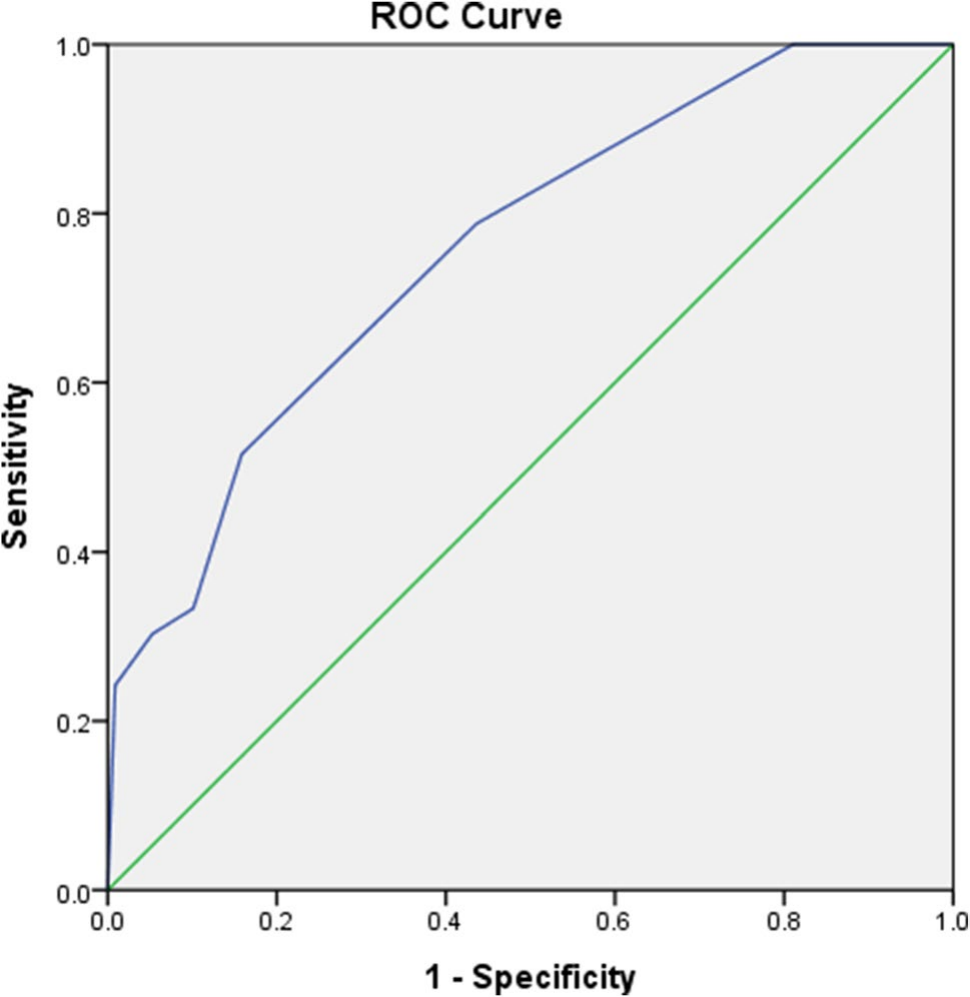
Receiver operator characteristic analysis of Distress Thermometer (DT) score for predicting suicidal ideation in patients with cancer

**Fig. 2:**
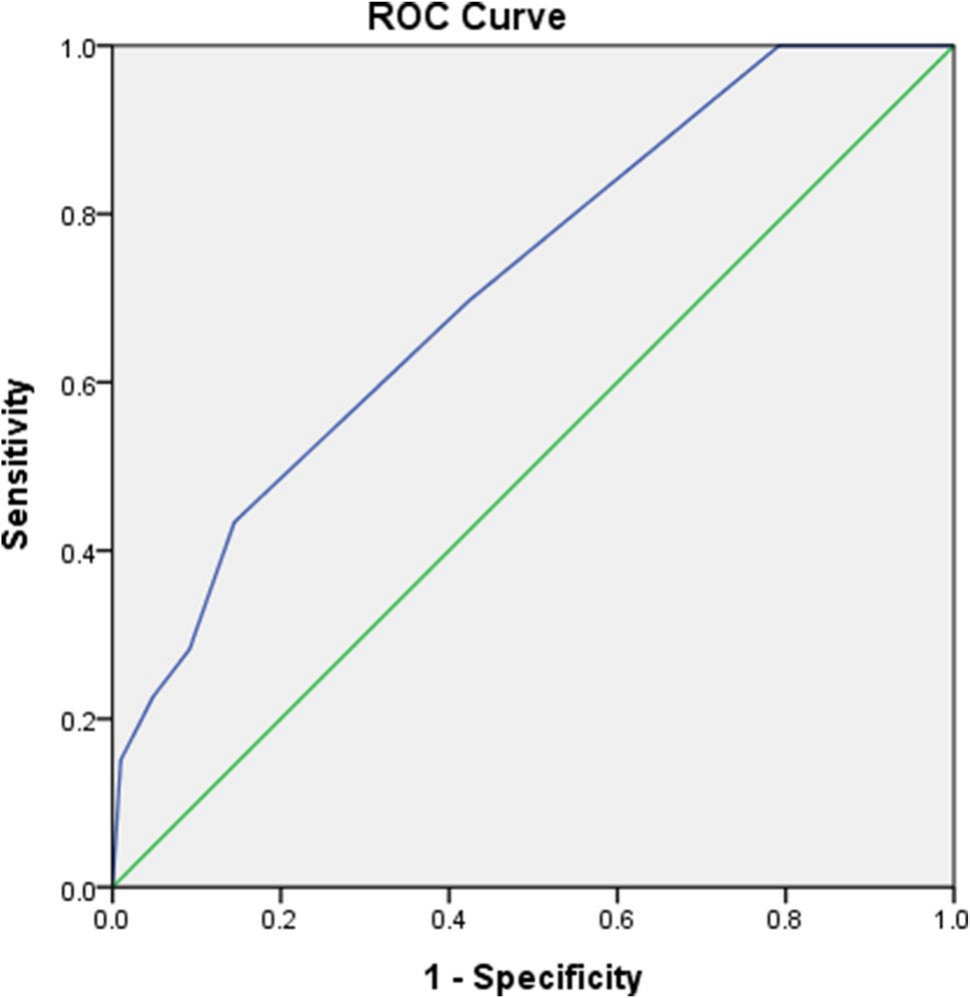
Receiver operator characteristic analysis of Distress Thermometer (DT) score for predicting death ideation in patients with cancer

Based on the ROC analysis for predicting patients with suicidal ideation, the AUC was 0.75 (Fig. 1). The appropriate cutoff DT score to predict patients with suicidal ideation was 5, with a sensitivity of 0.52, specificity of .84, LR^+^ of 3.24, and LR^-^ of 0.58 (Table 2). In addition, for patients with death ideation (including 33 patients with suicidal ideation), the AUC was 0.72 (Figure 2), and the appropriate cutoff DT score to predict patients with death ideation was 4, with a sensitivity of 0.70, specificity of 0.58, LR^+^ of 1.64, and LR^+^ of 0.53 (Table 3).

**Table 2:** Predictive power of Distress Thermometer (DT) score for different cutoff points in suicidal ideation group

DT score	Sensitivity	Specificity	LR^+^	LR^-^	Youden index
0	1.00	0.04	1.05	0	0.044
1.0	1.00	0.07	1.07	0	0.066
2.0	1.00	0.10	1.11	0	0.10
3.0	1.00	0.19	1.23	0	0.19
4.0	0.79	0.57	1.81	0.38	0.36
5.0*	0.52	0.84	3.24	0.58	0.36
6.0	0.33	0.90	3.30	0.74	0.23
7.0	0.30	0.95	5.72	0.74	0.25
8.0	0.24	0.99	26.89	0.77	0.23
9.0	0.12	1.00	30.25	0.88	0.12
10.0	0	1.00	-	1.00	0.000

*LR*^*+*^, likelihood ratio for a positive test; *LR*^*-*^, likelihood ratio for a negative test

^*^Best cutoff point

**Table 3:** Predictive power of Distress Thermometer (DT) score for different cutoff points in death ideation group

DT score	Sensitivity	Specificity	LR^+^	LR^-^	Youden index
0	1.000	0.05	1.05	0	0.05
1.0	1.000	0.07	1.08	0	0.07
2.0	1.000	0.11	1.13	0	0.11
3.0	1.000	0.21	1.26	0	0.21
4.0*	0.70	0.58	1.64	0.53	0.28
5.0	0.43	0.86	3.00	0.66	0.29
6.0	0.28	0.91	3.08	0.79	0.19
7.0	0.23	0.95	4.71	0.81	0.17
8.0	0.15	0.99	15.10	0.86	0.14
9.0	0.08	1.00	15.00	0.93	0.07
10.0	0	1.00	-	1.00	0

*LR*^*+*^, likelihood ratio for a positive test; *LR*^*-*^, likelihood ratio for a negative test

^*^Best cutoff point

We further determined two cutoff points for the DT score to “rule in” or “rule out” suicidal ideation and death ideation. The cutoff DT score of 3 had the highest sensitivity of 1.00 to rule out both suicidal ideation and death ideation, while 9 had the highest specificity of 1.00 to rule in both suicidal ideation and death ideation.

## Discussion

Suicide risk factors in patients with cancer includes the patient’s mental health, socio-demographic status, type of illness, site of cancer, physical functioning, and prognosis [16]. Because of the heterogeneity of suicidal behavior, it is still challenging for clinical staff to detect high-risk groups and provide early interventions. Asking about suicide can be difficult for staff other than mental health professionals. The literature reports the use of several screening tools to identify suicide risk in patients with cancer, including the Columbia-Suicide Severity Rating Scale (C-SSRS), National Institute of Mental Health Ask Suicide-Screening Questions (ASQ) Toolkit, the Distress Assessment and Response Tool (DART), Brief Symptom Inventory-18, and Patient Health Questionnaire-9 (PHQ-9) [2, 17, 18]. However, routine screening for suicide risk by using these screening tools for all cancer patients in clinical care could be challenging because of the burden on medical staff [19]; instead, it may be more feasible to use in selected group with higher risk.

Some studies have evaluated the relationship between depression and suicide in cancer patients and showed that depression is an important risk factor of suicide in patients with cancer [2, 20, 21]. Therefore, screening for depression is important in patients with cancer to reduce risk of suicide, and the Beck Depression Inventory, PHQ-9, and Hamilton Depression Rating Scale have been identified as reliable screening tools in this context [20]. In addition, demoralization was also proved to be highly related to suicide in patients with cancer and may also predict suicidality [22, 23]. Thus, identifying depression and demoralization in this patient population could help to screen for risk of suicide.

Although the DT is not a tool originally designed for assessing suicide risk, in our study, we found that it may be useful for preliminary screening suicide risk in patients with cancer. In this study, patients with suicidal ideation or death ideation had higher DT scores compared with those without suicidal and death ideation. Thus, we intended to gain insight into the sensitivity and specificity of DT scores for both suicidal and death ideation using ROC analysis. The AUC of ROC analysis for predicting patients with suicidal ideation and death ideation was 0.75 and 0.72, respectively, which showed acceptable discrimination, but not excellent. We determined a DT score of 5 as the optimal cutoff point to identify patients with suicidal ideation, and a DT score of 4 for death ideation. Clinically, once patients with cancer have a DT score greater than 4, the risk of death ideation will increase, while for a DT score greater than 5, the suicidal ideation risk will also increase. This finding is compatible with another study which suggested that a DT score of 5 as the cutoff point could be used for the preliminary screening of patients with cancer and high suicide risk [22].

DT score of 4 is the commonly recommended cutoff for a positive screen for distress in cancer patients. NCCN suggests further assessment to address patient’s distress if DT≥4 and recommends usual clinical care if DT≤3 [14]. In this study, we further found that a cutoff DT score of 3 had the highest sensitivity of 1.00 to rule out both suicidal ideation and death ideation, while 9 had the highest specificity of 1.00 to rule in both suicidal ideation and death ideation. Our findings suggest that clinicians need to pay more attention to patients with cancer where there is a DT score ≥9 because it indicates the highest risk of suicide. In contrast, patients with a DT score ≤3 may have very little risk of suicide. However, patients with a DT score between 4 and 8 should be closely followed and monitored by clinicians because of the uncertainty regarding suicide risk.

DT may not be regarded an ideal tool that accurately predict suicide risk in cancer patients; however, it might be able to initially rule in the patients with higher risk and rule out patients with low risk. This preliminary distinction of patients by DT score might reduce the burden from widespread suicide screening, and could be used as a reference while designing suicide prevention program in cancer care.

### Limitation

There are some limitations in this study. First, the research groups of inpatients only accounted for 1.17% of full population of admitted patients during the study period, and their referral to the psychologist may be due to some mental or emotional problems, including increased distress levels and higher DT scores. Thus, this patient group might not represent all the cancer patients admitted to our hospital, and the application of the results of this study to general cancer population may still need to be confirmed. Second, no suicide risk screening tool was used in this study. The suicide assessment was based on clinical assessment via a semi-structured interview. Although clinical assessment is widely used in real world practice, the comparison between clinical assessment and structured screening tool is lacking.

## Conclusion

The DT score may serve as a practical tool to screen for suicide risk in patients with cancer which can be incorporated easily into routine clinical care in all cancer settings. For a DT score greater than 5 in patients with cancer, the risk of suicide could increase. Most importantly, a cutoff DT score of 3 had the highest sensitivity of 1.00 to rule out suicidal ideation, while 9 had the highest specificity of 1.00 to rule in suicidal ideation. Future research is recommended to examine the correlation between the DT scores and suicidal ideation in general cancer population.

## Data Availability

Patient data was got through the information systems of Kaohsiung Veterans General Hospital.
